# Co‐localization of clinically relevant antibiotic‐ and heavy metal resistance genes on plasmids in *Klebsiella pneumoniae* from marine bivalves

**DOI:** 10.1002/mbo3.1368

**Published:** 2023-07-19

**Authors:** Fredrik Håkonsholm, Marit A. K. Hetland, Iren H. Löhr, Bjørn Tore Lunestad, Nachiket P. Marathe

**Affiliations:** ^1^ Institute of Marine Research Bergen Norway; ^2^ Department of Medical Biology, Faculty of Health Sciences University of Tromsø—The Arctic University of Norway Tromsø Norway; ^3^ Department of Medical Microbiology Stavanger University Hospital Stavanger Norway; ^4^ Department of Biological Sciences, Faculty of Mathematics and Natural Sciences University of Bergen Bergen Norway; ^5^ Department of Clinical Science, Faculty of Medicine University of Bergen Bergen Norway

**Keywords:** acquired antibiotic resistance, co‐selection, heavy metal resistance genes, *Klebsiella pneumoniae*, plasmids

## Abstract

*Klebsiella pneumoniae* is an opportunistic pathogen frequently associated with antibiotic resistance and present in a wide range of environments, including marine habitats. However, little is known about the development, persistence, and spread of antibiotic resistance in such environments. This study aimed to obtain the complete genome sequences of antibiotic‐resistant *K. pneumoniae* isolated from marine bivalves in order to determine the genetic context of antibiotic‐ and heavy metal resistance genes in these isolates. Five antibiotic‐resistant *K. pneumoniae* isolates, of which four also carried heavy metal resistance genes, were selected for complete genome sequencing using the Illumina MiSeq platform and the Oxford Nanopore Technologies GridION device. Conjugation experiments were conducted to examine the transfer potential of selected plasmids. The average length of the complete genomes was 5.48 Mbp with a mean chromosome size of 5.27 Mbp. Seven plasmids were detected in the antibiotic‐resistant isolates. Three IncFIB, one IncFIB/IncFII, and one IncFIB/IncHIB plasmid, respectively, carried antibiotic resistance genes such as *qnrS1, aph(6)‐Id* and *aph(3′)‐Ia, aadA1*, and *aadA2*. Four of these plasmids also carried genes encoding resistance to copper (*pco*), silver (*sil*), and arsenic (*ars*). One plasmid carrying *tet(D*) and *bla*_SHV‐1_ as well as *pco, sil*, and *ars* genes was transferred to *Escherichia coli* by conjugation. We show the co‐occurrence of antibiotic‐ and heavy metal resistance genes on a conjugative IncFIB plasmid from *K. pneumoniae* from marine bivalves. Our study highlights the importance of the marine environment and seafood as a possible dissemination route for antimicrobial resistance and provides insights into the potential for co‐selection of antibiotic resistance genes by heavy metals.

## Graphical abstract

*Klebsiella pneumoniae* is an opportunistic pathogen frequently associated with antibiotic resistance and present in a wide range of environments. In this study, five antibiotic‐resistant *K. pneumoniae* isolates recovered from bivalves, of which four also carried heavy metal resistance genes, were selected for complete genome sequencing. This study shows the co‐occurrence of antibiotic‐ and heavy metal resistance genes on a transferable IncFIB plasmid from *K. pneumoniae* from marine bivalves and further highlights the importance of the marine environment and seafood as a possible dissemination route for antimicrobial resistance.

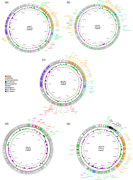

## 1 INTRODUCTION

*Klebsiella pneumoniae* is an opportunistic pathogen and a common cause of nosocomial infections. *K. pneumoniae* is often associated with antibiotic resistance, and strains resistant to clinically important antibiotics are considered a critical threat to public health (Wyres et al., 2020). *K. pneumoniae* is commonly found in the gastrointestinal tract of humans and animals but can also be isolated from a range of environments, including soil, plants, surface waters, and marine organisms (Brisse et al., 2006; Håkonsholm et al., 2022; Wyres et al., 2020).

Increased resistance to antibiotics is one of the greatest threats in modern medicine (Church & McKillip, 2021). Infections caused by antibiotic‐resistant bacteria were estimated to be the direct cause of 1.27 million deaths globally in 2019 (Murray et al., 2022). *K. pneumoniae* is considered an important contributor to the spread of antibiotic resistance (Wyres & Holt, 2018). Resistance to broad‐spectrum cephalosporins and carbapenems is increasingly reported in clinical *K. pneumoniae* isolates in the WHO European region, with 44% of countries reporting resistance rates of ≥50% to third‐generation cephalosporins in 2020, particularly in southern and eastern European countries. However, the occurrence is still low in Scandinavian countries with an average of 8.1% invasive *K. pneumoniae* isolates resistant to third‐generation cephalosporins (WHO Regional office for Europe, ECDC, 2021).

Horizontal gene transfer is one of the primary drivers of antibiotic resistance, and the spread of antibiotic‐resistance genes (ARGs) is driven by conjugative plasmids (San Millan, 2018). Plasmids can be classified according to their incompatibility (Inc), which refers to their inability to co‐exist stably in the same cell line over time. In general, closely related plasmids are often incompatible, while those more distantly related often are compatible. Overall, 28 Inc groups have been reported within the Enterobacterales family, and some of these are frequently associated with ARGs, for example, extended‐spectrum β‐lactamase (ESBL) and carbapenemase‐encoding genes are commonly found on IncF plasmids (Carattoli, 2009; Rozwandowicz et al., 2018). In *K. pneumoniae*, most of the ARGs are present on large conjugative plasmids, and most acquired ARGs are carried on plasmids belonging to the IncFII, IncN, IncR, and/or IncX3 groups (Wyres & Holt, 2018; Wyres et al., 2020).

The environment is recognized as important habitat for the development and spread of antibiotic resistance (Bengtsson‐Palme et al., 2017; Marathe et al., 2017). Although overuse of antibiotics is a major driver of antibiotic resistance, other compounds such as heavy metals and biocides can cause co‐selection of antibiotic‐resistant bacteria. Unlike antibiotics, metals in the environment are not degraded, and their presence could therefore represent a long‐term selection pressure (Baker‐Austin et al., 2006).

In a previous study, we have shown the presence of antibiotic‐resistant *K. pneumoniae* carrying heavy metal resistance genes (HMRGs) in bivalve mollusks and seawater from the Norwegian marine environment (Håkonsholm et al., 2022). The present study aimed to obtain complete genome sequences of *K. pneumoniae* isolates carrying ARGs and HMRGs using a combination of long‐ and short‐read whole‐genome sequencing in order to determine the genetic context of ARGs and HMRGs in this setting. We show the co‐occurrence of ARGs and HMRGs on a conjugative IncFIB plasmid in one of the isolates.

## 2 MATERIALS AND METHODS

### 2.1 Bacterial isolates

Five *K. pneumoniae* sensu stricto isolates with acquired ARGs recovered from marine bivalves were selected for complete genome sequencing (Håkonsholm et al., 2022). Four isolates were recovered from blue mussels (*Mytilus edulis*) and one from oysters (*Crassostrea gigas*). Antibiotic susceptibility testing of the isolates was done by disk diffusion as described previously (Håkonsholm et al., 2020). Measured inhibition zones were interpreted according to EUCAST breakpoints for Enterobacterales (https://www.eucast.org/clinical_breakpoints).

### 2.2 Whole‐genome sequencing, hybrid de novo assembly, and bioinformatic analyses

The short‐read sequencing was performed as described previously (Håkonsholm et al., 2022). For the long‐read sequencing, DNA was extracted manually using the Beckman Coulter Life Science GenFind V3 with the protocol: “DNA extraction from Bacteria using GenFindV3” (Beckman Coulter). Library preparation was done with the SQK‐LSK‐109 kit (Oxford Nanopore Technologies), DNA libraries were loaded onto a MINion flow cell (R9.4.1), and sequencing was done using the Oxford Nanopore Technologies GridION device. Base‐calling was performed with Guppy v4.2.2 + effbaf84 (https://community.nanoporetech.com) and quality filtering using FiltLong v0.2.0 (https://github.com/rrwick/Filtlong).

Hybrid de novo assembly of the short‐ and long‐read sequences was done with Unicycler v0.4.8 (Wick et al., 2017). The assemblies were analyzed with AMRFinder plus v3.9.8 (Feldgarden et al., 2019) and PlasmidFinder v2.1 (database version 2021‐11‐29) (Carattoli et al., 2014). The sequence identities of ARGs, HMRGs as well as plasmid replicons, and the coverage of these against their references are provided in Supporting Information: Table S1. The assembled genomes were annotated through the NCBI prokaryotic genome annotation pipeline v5.3 (Tatusova et al., 2016). Both the assembled and annotated genomes were screened for virulence factors using the VFanalyzer available through the Virulence Factor Database (VFDB) (http://www.mgc.ac.cn/cgi-bin/VFs/v5/main.cgi) (Chen et al., 2016). BLASTN v2.13.0+ (Camacho et al., 2009) was used to compare the plasmid sequences to previously described plasmids. Circular plasmid maps were created using the Proksee server (https://proksee.ca) and alignments of plasmid regions carrying HMRGs were generated with Easyfig v2.2.5 (Sullivan et al., 2011) using a minimum sequence identity of 80%.

Previously published Illumina reads from isolates carrying the same HMRGs (*sil, pco*, and *ars* [*n* = 13], or *sil, pco, ars*, and *mer* [*n* = 2]) and IncFIB plasmid replicons were mapped against the complete plasmid sequences generated for the present study using the RedDog pipeline (https://github.com/katholt/RedDog) (Håkonsholm et al., 2022). Single‐nucleotide polymorphism (SNP) matrices were generated with SNP‐dists v0.7.0 (https://github.com/tseemann/snp-dists). The criteria proposed by Hawkey et al. (2022) (>80% mapping cover and <10 SNPs) were used to determine the presence of the closed plasmids in draft genomes. Mapping statistics and SNP matrices are available in Supporting Information: Tables S2 and S3, respectively.

### 2.3 Conjugation experiments

*K. pneumoniae* isolates 2016‐319 and 2016‐1200 carried resistance plasmids encoding multiple genes involved in the conjugal transfer and were subjected to conjugation experiments by filter‐mating following a previously described method (Jutkina et al., 2016). Kanamycin (KAN) and rifampicin (RIF) resistant green fluorescent protein (GFP) tagged *E. coli* CV601‐GFP strain was used as the recipient. The recipient strain was grown overnight in Muller Hinton broth (MHB) (Oxoid) supplemented with 50 µg/mL KAN (Glentham Life Sciences) at 30°C with shaking (200 rpm), while the donors were grown in MHB supplemented with 100 µg/mL ampicillin (AMP) (Sigma‐Aldrich) in the same conditions. Donor and recipient mixtures were washed twice in phosphate‐buffered saline (PBS) (Sigma‐Aldrich) and mixed at a ratio of 1:1 before filtering through 0.45 µm pore filters and placing the filter on Mueller Hinton plates (Oxoid). The plates were incubated aerobically at 30°C for 3 h, and the mating was disrupted by vortexing the filters in tubes with 10 mL PBS and sterile glass beads. Serial dilutions were prepared in PBS and 100 µL of the 10^−2^ and 10^−3^ dilutions were spread on CHROMagar Orientation plates (CHROMagar) supplemented with KAN (50 µg/mL), RIF (50 µg/mL) (Glentham Life Sciences), and AMP (100 µg/mL) and incubated at 35°C for ~36 h. The antibiotic sensitivity patterns of transconjugants were examined by disk diffusion following the EUCAST method (Matuschek et al., 2014). Transfer frequencies were calculated using the total number of recipients before mating.

## 3 RESULTS

### 3.1 Complete genome sequences of *K. pneumoniae* isolates encoding ARGs and HMRGs

**Figure A1:**
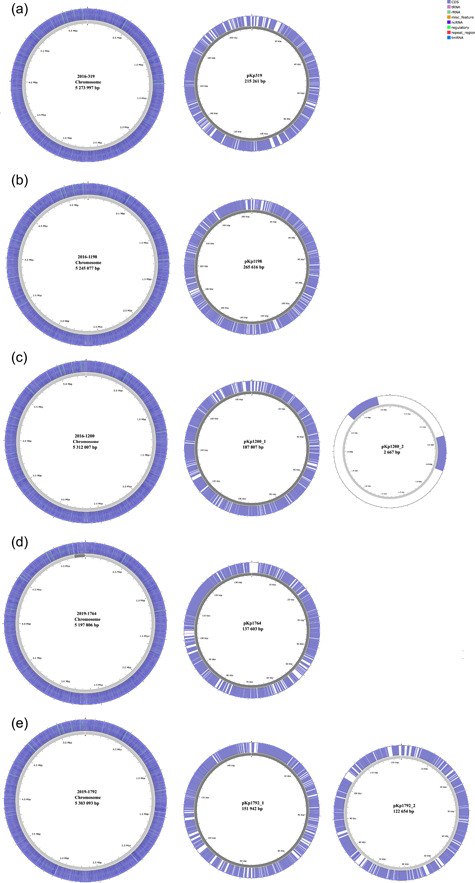
Complete genomes of *Klebsiella pneumoniae* isolated from marine bivalves. (a) Isolate 2016‐319, (b) isolate 2016‐1198, (c) isolate 2016‐1200, (d) isolate 2019‐1764, (e) isolate 2019‐1792.

The size of the assembled genomes ranged from 5.34 to 5.58 Mbp, with a mean GC content of 57.3% (57.1%–57.4%) and an average chromosome size of 5.27 Mbp (5.20–5.31 Mbp). Overall, seven plasmids were identified in the assembled genomes, ranging in size from 2667 to 265,616 bp (Figure A1). Five plasmids, identified in five separate isolates, carried acquired ARGs, while four of these also carried genes encoding resistance to heavy metals. Phenotypic antibiotic susceptibility data for the isolates included in this study are provided in Supporting Information: Table S4. All acquired ARGs and HMRGs were co‐located on IncFIB plasmids (Table 1).

**Table 1:** Complete genome sequences of *Klebsiella pneumoniae* isolates included in the study, acquired antibiotic resistance genes (ARGs), heavy metal resistance genes (HMRGs), acquired virulence genes, and plasmid replicons.

Isolate	Sequence type	Contig	Size (bp)	Accession no.	ARGs	HMRGs	Virulence genes/loci	Plasmid replicons
2016‐319	ST556	Chromosome	5,273,997	CP085101	–	–	–	–
		pKp319	215,261	CP085102	*tet(D), bla*_SHV‐1_	*silABCEFPRS, pcoABCDERS, arsABCDHR* a	–	IncFIB(K)
2016‐1198	ST2167	Chromosome	5,245,077	CP085097	–	–	–	–
		pKp1198	265,616	CP085098	*tet(D), catA2, sul2*	*silABCEFPRS, pcoABCDERS, arsBCH, merACDEFPRT* b	–	IncFIB(K)(pCAV1099‐114), IncHI1B(pNDM‐MAR)
2016‐1200	ST25	Chromosome	5,312,007	CP085033	–	–	*ybt*	–
		pKp1200_1	187,807	CP085034	*sul1, aph(3′)‐Ia, dfrA14, tet(D), bla*_TEM‐1_, *aph(6)‐Id, aph(3″)‐Ib sul2*	*silABCEFPRS, pcoABCDERS, arsABCDHR*	–	IncFIB(K), IncFII(K)
		pKp1200_2	2667	CP085035	–	–	–	–
2019‐1764	ST292	Chromosome	5,197,806	CP085099	–	–	–	–
		pKp1764	137,603	CP085100	*dfrA12, aadA1, aadA2, cmlA1, bla*_TEM‐1_, *qnrS1, sul3*	–	–	IncFIB(pKPHS1)
2019‐1792	ST4267	Chromosome	5,303,093	CP085103	–	–	–	–
		pKp1792_1	151,942	CP085104	–	–	–	IncFII(K)
		pKp1792_2	122,654	CP085105	*tet(A)*	*silABCEFPRS, pcoABCDERS, arsABCDR*	*mrkABCDFJIH*	IncFIB(K)

^a^ pKp319 carried two copies of *arsH, arsC, arsB*, and one truncated copy of *arsA*.

^b^ pKp1198 carried two copies of *merT*, *merP, merR*, and one truncated copy of *m*.

**Figure 1:**
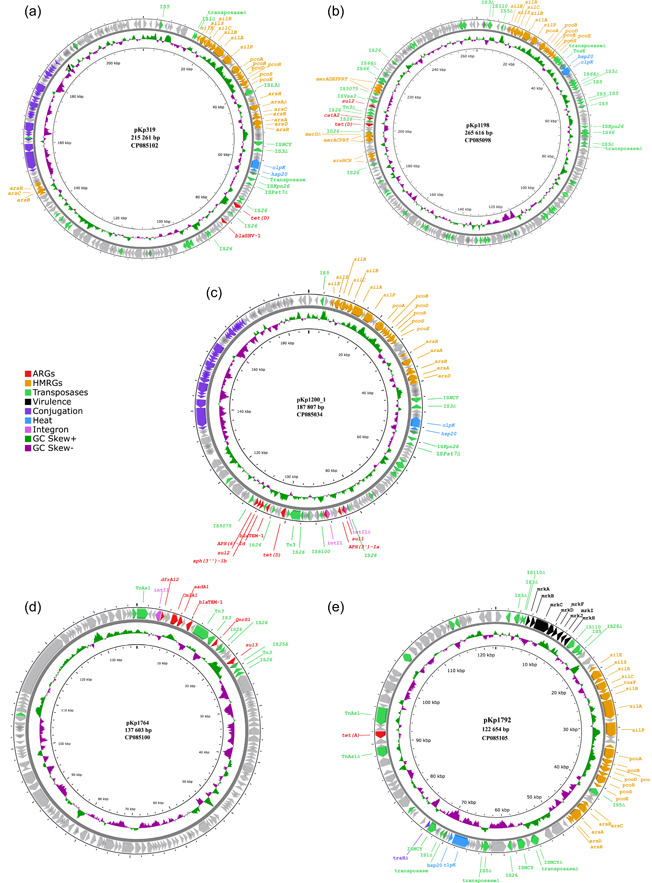
Genomic maps of the resistance plasmids from *K. pneumoniae* isolated from the marine environment. (a) pKp319, (b) pKp1198, (c) pKp1200_1, (d) pKp1764, and (e) pKp1792_2. Genes are colored according to function: Red, antibiotic resistance genes (ARGs); orange, heavy metal resistance genes (HMRGs); green, transposases; blue, heat tolerance; purple, conjugation; pink, Integrons; black, virulence. GC skew is indicated in green and purple. Truncated genes are indicated with ∆. ARGs, HMRGs, and the transposases associated with them are labeled.

*K. pneumoniae* ST556 isolate 2016‐319 (CP085101) was recovered from a pooled sample of *C. gigas* collected from a commercial production location and carried IncFIB plasmid pKp319 (CP085102), which encoded all the acquired ARGs and HMRGs detected in the strain. Plasmid pKp319 carried tetracycline‐ (*tet(D)*) and penicillin resistance genes (*bla*_SHV‐1_) within a 13,867 bp (73,169–87,036 bp) region flanked by IS*26* transposases. Furthermore, plasmid pKp319 carried copper (*pco*), silver (*sil*), and arsenic resistance genes (*ars*), as well as genes related to heat tolerance (*clpK, hsp20*) in a region flanked by IS*5* transposases (IS*903* and IS*Kpn26*). Plasmid pKp319 also carried additional copies of the *arsB*, *arsC*, and *arsH* genes (Figure 1a). Additionally, this plasmid encoded a Type II toxin‐antitoxin (TA) system (RelE/ParE family toxin, Phd/YefM family antitoxin) involved in plasmid maintenance (Kamruzzaman et al., 2021). Comparing plasmid pKp319 to other publicly available plasmid sequences, it was identical to plasmid tig00001208_pilon (CP036443) (99% coverage and >99.9% identity) from a clinical *K. pneumoniae* ST45 strain ABFPV (CP036442) from the USA isolated in 2014. However, plasmid pKp319 carried accessory regions, including a 3 337 bp region encoding the betT choline transporter, as well as a 4329 bp region carrying plasmid maintenance genes like *psiB* and an additional copy of the ParB/RepB/Spo0J family partition protein‐coding genes, absent in plasmid tig00001208_pilon.

IncFIB/IncHI1B plasmid pKp1198 (CP085098), identified in *K. pneumoniae* ST2167 isolate 2016‐1198 (CP085097) was recovered from a rearing facility in northern Norway, and carried genes encoding resistance to tetracycline (*tet(D)*), sulfonamides (*sul2*), chloramphenicol (*catA2*), mercury (*merACDEPRT*), additional copies of *merAPRT*, a truncated copy of *merD* and *arsBCH* on an ~43,200 bp region (185,828–229,073 bp) flanked by IS*26* transposases. In plasmid pKp1198, the *pco* and *sil* locus, *clpK* and *hsp20* were present in a region flanked by truncated and complete IS*5* family transposases (Figure 1b). Furthermore, plasmid pKp1198 carried multiple Type II TA systems (HigB family toxin, VapC family toxin, VapB family antitoxin, and phd/YefM). This plasmid was highly similar (98% coverage and >99.9% identity) to plasmid p59062CZ_IncFIB (CP085732) encoding resistance to tetracycline (*tet(D)*) and chloramphenicol (*catA1*) from a clinical *K. pneumoniae* ST54 strain isolated in the Czech Republic in 2020. Both plasmids carried ARGs and HMRGs; however, pKp1198 carried *sul2* and *merDEF* within this region which is absent in plasmid p59062CZ_IncFIB. Also, plasmid pKp1198 harbored other accessory genes in a region ~8000 bp in size, encoding the *fec* system (*fecIRABCDE*) flanked by IS*100* transposases.

*K. pneumoniae* ST25 isolate 2016‐1200 (CP085033) was isolated from *Mytilus edulis* collected from a commercial production location. This isolate carried all ARGs and HMRGs on IncFIB/IncFII plasmid pKp1200_1 (CP085034). The ARGs were located within an ~28 500 bp (82,483–110,975 bp) region, which contained genes encoding resistance to sulfonamides (*sul1, sul2*), aminoglycosides (*aph(3′)‐Ia, aph(3″)‐Ib, aph(6)‐id*), tetracycline (*tet(D)*), trimethoprim (*dfrA14*), and penicillins (*bla*_TEM‐1_). Within the region, one complete and one truncated class 1 integron with gene cassettes carrying *sul1, aph(3′)‐Ia*, and *dfrA14* were identified. All ARGs were located in a region flanked by IS*26* and IS*5075* transposases. Similar to plasmid pKp319, plasmid pKp1200_1 carried multiple genes encoding resistance to silver (*sil*), copper (*pco*), and arsenic (*ars*), in addition to *clpK* and *hsp20* on an ~48,000 bp region flanked by IS*5* transposases (Figure 1c). Overall, plasmid pKp319 from isolate 2016‐319 and plasmid pKp1200_1 from isolate 2016‐1200 shared 76% of the nucleotide sequence with >99% identity. Plasmid pKp1200_1 carried the RelE/ParE and phd/YefM Type II TA system. BLAST searches against publicly available plasmid sequences showed that plasmid pKp1200_1 has a similar backbone to several other plasmids detected in *K. pneumoniae* (>80% sequence coverage, >99% identity).

*K. pneumoniae* ST292 isolate 2019‐1764 (CP085099) was isolated from *M. edulis* collected from an area used for recreational activities and was the only antibiotic‐resistant isolate lacking HMRGs. IncFIB plasmid pKp1764 (CP085100) carried all ARGs on a region located between position 1 and 23,546 bp. A class 1 integron with a gene cassette containing ARGs encoding resistance to trimethoprim (*dfrA12*), aminoglycosides (*aadA1, aadA2*), and chloramphenicol (*cmlA1*) was identified within this region. Furthermore, pKp1764 harbored genes encoding resistance to penicillins (*bla*_TEM‐1_) on a Tn*3* transposon and also carried *qnrS1* and *sul3* (Figure 1d) encoding resistance to quinolones and sulfonamides, respectively. Several phage‐related genes were identified in the plasmid sequence, and the backbone of plasmid pKp1764 was similar (80% coverage, 99.97% identity) to the phage‐like pSID3 plasmid (CP066514), from the clinical *K. pneumoniae* ST893 strain Kp36336 (CP066511) isolated from a Belgian patient in 2019, and an unnamed *K. pneumoniae* plasmid (CP063431) of human origin reported from Singapore (Eskenazi et al., 2022). However, the resistance region on plasmid pKp1764 was absent on plasmid pSID3 and CP063431, but identical (100% sequence coverage, >99% identity) to segments present on the chromosome of two *Escherichia coli* strains recovered from pork in China (CP037903) in 2017 and Cambodia (CP044291) in 2016. The resistance region on plasmid pKp1764 was also similar (>99% identity) to segments in an *E. coli* plasmid recovered from wastewater in the UK (CP056847) in 2017 which also carries a similar class 1 integron (92% coverage, >99% identity).

*K. pneumoniae* isolate 2019‐1792 (CP085103) belonging to ST4267 carried two plasmids, IncFII plasmid pKp1792_1 (CP085104) and IncFIB plasmid pKp1792_2 (CP085105), of which plasmid pKp1792_2 carried the *tet(A)* tetracycline resistance gene on a *TnaS1* transposable element (Figure 1e). Similar to other plasmids identified in *K. pneumoniae* from marine bivalves collected in Norway, plasmid pKp1792_2 harbored the *sil, pco*, and *ars* genes, the *clpK* and *hsp20* genes, as well as a Type II TA system (RelE/ParE, phd/YefM). Plasmid pKp1792_2 also carried the *mrkABCDFJIH* fimbriae genes flanked by IS*110* transposases that are also present on the chromosome of *K. pneumoniae* (Wyres et al., 2020). BLASTn analysis showed that plasmid pKp1792_2 was similar (86% sequence coverage and 99.9% identity) to plasmid pK039_3 (CP034362) from a *K. pneumoniae* ST403 isolate (CP034359) from a carrier in Tanzania.

Identical plasmids could not be identified in the draft genomes of other isolates from our collection carrying the same HMRGs as well as the IncFIB(K) replicon type, but lacking acquired ARGs (Håkonsholm et al., 2022). Only *K. pneumoniae* isolates ST337 2020‐586 and ST337 2020‐584/2 showed the presence of plasmid similar to pKp1792_2 (>90% sequence coverage and >99% identity), while *K. pneumoniae* isolates ST220 2016‐729 and ST39 2019‐400/1 showed the presence of plasmids similar to pKp319 (>80% sequence coverage with >99% identity).

### 3.2 Comparison of plasmid regions carrying HMRGs

**Figure 2:**
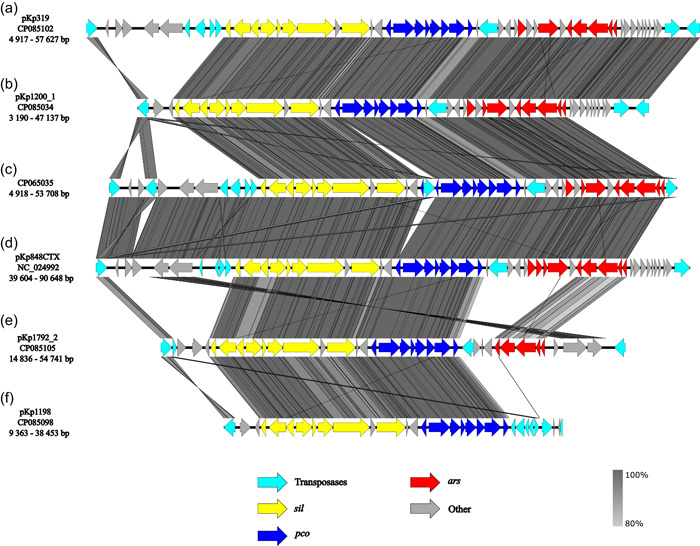
Alignment of plasmid regions carrying heavy metal resistance genes. (a) position 4917 to 57,627 bp of plasmid pKp319 (CP085102), (b) position 3190–47,137 bp of plasmid pKp1200_1 (CP085034), (c) position 4918– 53,708 bp of plasmid CP065035, (d) position 39,604–90,648 bp of plasmid pKp848CTX (NC_024992), (e) position 14,836–54,741 bp of plasmid pKp1792 (CP085105), (f) position 9363–38,453 of plasmid pKp1198 (CP085098). The *Sil* operon is highlighted in yellow, the *pco* operon is colored blue, *ars* genes are colored red, and insertion sequences and transposases are highlighted in cyan. Other genes present in the region are colored gray.

The HMRGs carrying regions of the different plasmids all carried the *pco* and *sil* operon in similar regions but flanked by different transposases. Similar regions were also identified in previously published plasmid CP065035 from *M. edulis* and the *bla*_CTX‐M‐15_ encoding plasmid pKp848CTX from *K. pneumoniae* ST17 (Kp848) causing an outbreak at Stavanger University Hospital in 2009 (Löhr et al., 2015) (Figure 2). In pKp1200_1 and pKp319 *sil* and *pco* genes were located together with the *ars* genes as well as genes related to heat tolerance in a region flanked by IS*5* family transposases, similar to plasmid CP065035 (Håkonsholm et al., 2020). In plasmid pKp1792_2, the *sil, pco, ars, clpK*, and *hsp20* genes were flanked by an IS*5* transposase, whereas plasmid pKp1198 carried the *sil* and *pco* operon and heat tolerance genes in a region containing several different transposases and *ars* and *mer* were clustered with ARGs in a separate region flanked by IS*26*.

### 3.3 Conjugation assay

Plasmid pKp319 from *K. pneumoniae* isolate 2016‐319 was transferred to *E. coli* CV601‐GFP strain, yielding transconjugants with identical resistance patterns (AMP^R^, TET^R^) at a transfer frequency of 5.1 ×  10^−4^ transconjugants per recipient cell. Antibiotic susceptibility patterns of the obtained transconjugants are provided in Supporting Information: Table S1. Even though we predicted plasmid pKp1200_1 to be conjugative based on the genotype, we were not able to verify the conjugative transfer of this plasmid to the *E. coli* recipient in repeated experiments, indicating either a very low transfer frequency or inability of pKp1200_1 to transfer to *E. coli*.

## 4 DISCUSSION

In the present study, we report the complete genome sequences of antibiotic‐resistant *K. pneumoniae* isolated from bivalve mollusks collected along the Norwegian coast. We show the co‐localization of ARGs and HMRGs on IncF plasmids, suggesting the potential for co‐selection of ARGs by heavy metals in the marine environment.

Five of the identified plasmids carried genes encoding resistance to aminoglycosides, sulfonamides, cephalosporins, tetracycline, quinolones, and/or amphenicols, all considered to be important by the WHO for the treatment of infections in humans (World Health Organization, 2019). Most of the plasmids carrying ARGs belonged to the IncF group. IncF is the most frequently described plasmid type, and is commonly found in bacteria of both human and animal origin (Rozwandowicz et al., 2018). Previous studies have also shown that IncF plasmid replicons are common in Norwegian *K. pneumoniae* isolates from clinics, wastewater, and from community‐based carriers (Fostervold et al., 2021; Radisic et al., 2023; Raffelsberger et al., 2021). Furthermore, a study on antibiotic‐resistant *E. coli* in marine sediments and clams collected in Italy found IncF type plasmids as the most common plasmid type carrying ARGs, while another study found IncF plasmids among CTX‐M producing *E. coli* and *K. pneumoniae* isolated from marine bivalves in Brazil, consistent with our results for *K. pneumoniae* (Bueris et al., 2022; Citterio et al., 2020). Plasmids belonging to this Inc group are often associated with ARGs and are recognized as important contributors to the spread of antibiotic resistance, especially quinolone resistance genes, ESBLs, carbapenemases, and genes encoding resistance to aminoglycosides (Rozwandowicz et al., 2018). This is in accordance with our results, where three of the five resistance plasmids carried genes involved in resistance to quinolones, aminoglycosides, or β‐lactam antibiotics, suggesting that such plasmids may be important in the dissemination of ARGs also in the marine environment.

Only plasmid pKp319 encoding resistance to tetracycline and ampicillin was transferred to the *E. coli* recipient via conjugation. The presence of ARGs and HMRGs on a conjugative plasmid indicates the potential for dissemination of such plasmids in the marine environment. However, we were not able to show the transmissibility of plasmid pKp1200_1, which carried multiple ARGs and genes related to conjugation to the recipient used in this study. This is in accordance with a previous study showing the inability of a CTX‐M encoding IncFIB(K)/IncFII(K) plasmid (pKp848CTX), carrying a conserved transfer region, to transfer to an *E. coli* recipient in broth and filter mating experiments (Löhr et al., 2015). Further experiments using recipients belonging to different species/genera, including other *Klebsiella* spp., may be necessary to confirm the transferability of plasmid pKp1200_1. Additionally, pKp1200_1 carried genes encoding Klebicin B, a bacteriocin with nuclease activity, possibly reducing the number of recipient cells (Riley & Wertz, 2002; Riley et al., 2001).

Even though the plasmids reported in this study carry clinically relevant ARGs, one of the important findings of our study is the co‐localization of ARGs and HMRGs on the same plasmids in *K. pneumoniae* isolated from marine bivalves. HMRGs are frequently reported in clinical *K. pneumoniae* isolates as well as isolates from wastewater and marine environments, including seafood organisms (Furlan et al., 2020; Håkonsholm et al., 2022; Radisic et al., 2023; Sütterlin et al., 2017). Interestingly, most plasmids included in the present study carried similar regions harboring the *sil* and *pco* operon, and also genes encoding heat tolerance (*hsp20, clpK*), indicating that this region could be common in *K. pneumoniae* plasmids belonging to the IncFIB group. Furthermore, in plasmids pKp319, pKp1200_1, CP065035, and pKp848CTX the *sil* and *pco* operons were flanked by *IS*5 and truncated *IS*L3 transposases, possibly indicating that this composite transposon is important in the dissemination of heavy metal resistance, also in clinical settings (Löhr et al., 2015; Sütterlin et al., 2017). Overall, HMRGs were associated with different types of transposases in all plasmids, including members of the IS*5* and IS*26* families, indicating a potential for mobilization of these genes (Partridge et al., 2018).

Norway has a low prevalence of antibiotic resistance and low use of antibiotics in both human and veterinary medicine (NORM/NORM‐VET, 2020). However, heavy metals, such as copper, are used in the aquaculture industry, both in antifouling agents and as additives in fish feed (Grefsrud et al., 2021; Seiler & Berendonk, 2012). As a result, copper can contaminate the marine environment through fecal material, spilled feed, and leakage from metal‐impregnated fish farm nets (Grefsrud et al., 2021). Copper is also naturally occurring in marine sediments and seawater (Grefsrud et al., 2021). Additionally, metal compounds, including arsenic, are used in wood preservation, livestock feed, and in agriculture as pesticides, fertilizers, and antimicrobials (Pal et al., 2017; Seiler & Berendonk, 2012; Silbergeld & Nachman, 2008), and can therefore be spread to the marine environment through run‐off from agricultural land. It has been suggested that *pcoA* and *pcoB* alone can confer copper resistance; however, *pcoC, pcoD*, and *pcoE* are required for full copper resistance (Argudín et al., 2019). The plasmids detected in our study harbored *pcoABCDE*, *silABCEFPRS*, and arsenic resistance genes. Previously, Gullberg et al. (2014) have shown that low concentrations of copper and especially arsenic were sufficient to maintain the multi‐drug resistance pUUH239.2 plasmid (NC_016966), carrying *ars* and *pco* genes, from a *K. pneumoniae* strain responsible for a nosocomial outbreak in Sweden. Thus, our results indicate the potential for co‐selection of ARGs in *K. pneumoniae* in metal‐contaminated marine environments.

Furthermore, all plasmids encoding both ARGs and HMRGs characterized in the present study carried type II TA systems, responsible for the killing, or growth‐inhibition, of plasmid‐free progeny cells (Kamruzzaman et al., 2021). These TA systems thus ensure that the plasmids are maintained and disseminated in bacterial populations even in environments without selection pressure imposed by antibiotics and/or heavy metals (Martinez, 2009). These plasmids can potentially persist in such environments and be transferred to human microbiota through, for example, seafood or direct contact via recreational activities.

## 5 CONCLUSION

In the present study, we report the complete genome sequences of antibiotic‐resistant *K. pneumoniae* isolated from marine bivalve mollusks collected along the Norwegian coast. We show co‐localization of ARGs and HMRGs on IncFIB, IncFIB/IncFII, and IncFIB/IncHIB plasmids present in *K. pneumoniae* isolated from the marine bivalves. We further show that one of the plasmids carrying ARGs and HMRGs is transferrable to *E. coli* via conjugation. Our study shows the potential for co‐selection of ARGs and/or antibiotic‐resistant *K. pneumoniae* in the marine environment by heavy metals. It also demonstrates the importance of the marine environment and seafood as dissemination routes for ARGs and pathogens and highlights the need for surveillance of antibiotic resistance in the marine environment.

## AUTHOR CONTRIBUTIONS

**Fredrik Håkonsholm**: Conceptualization (supporting); data curation (equal); formal analysis (lead); investigation (lead); methodology (lead); visualization (lead); writing—original draft (lead). **Marit A. K. Hetland**: Data curation (equal); formal analysis (equal); writing—review & editing (equal). **Iren H. Löhr**: Conceptualization (equal); funding acquisition (lead); project administration (equal); writing—review & editing (equal). **Bjørn Tore Lunestad**: Conceptualization (equal); project administration (equal); supervision (equal); writing—review & editing (equal). **Nachiket P. Marathe**: Conceptualization (equal); funding acquisition (equal); methodology (equal); project administration (lead); supervision (equal); validation (lead); writing—review & editing (equal).

## CONFLICT OF INTEREST STATEMENT

The authors declare no conflict of interest.

## ETHICS STATEMENT

None required.

## Supporting information

Supporting information.

## Data Availability

Genome assemblies and annotations are available in GenBank under BioProject PRJNA769247: https://www.ncbi.nlm.nih.gov/bioproject/PRJNA769247. GenBank accession numbers for individual genomes and plasmids are presented in Table 1.
